# Potential Efficacy of Erythropoietin on Reducing the Risk of Mortality in Patients with Traumatic Brain Injury: A Systematic Review and Meta-Analysis

**DOI:** 10.1155/2020/7563868

**Published:** 2020-10-29

**Authors:** Chengli Liu, Changsheng Huang, Jie Xie, Hui Li, Michael Hong, Xuemei Chen, Junmin Wang, Jiarui Wang, Zhanfei Li, Jian Wang, Wei Wang

**Affiliations:** ^1^Department of Traumatic Surgery, Tongji Hospital, Tongji Medical College, Huazhong University of Science and Technology, Wuhan 430030, China; ^2^Department of Gastrointestinal Surgery, Tongji Hospital, Tongji Medical College, Huazhong University of Science and Technology, Wuhan 430030, China; ^3^Maryland University School of Medicine, Baltimore, MD 21201, USA; ^4^Department of Anatomy, College of Basic Medical Sciences, Zhengzhou University, Henan 450000, China; ^5^The Johns Hopkins University, Baltimore, MD 21218, USA

## Abstract

**Objective:**

The objective of this study is to assess the effectiveness of erythropoietin (EPO) on mortality, neurological outcomes, and adverse event in the treatment of traumatic brain injury (TBI).

**Methods:**

We searched databases including PubMed, OVID, and the Cochrane Library from inception until October 18, 2019 for randomized controlled trials (RCTs) to compare EPO treatment group and placebo in patients with TBI. Two authors independently processed the data and evaluated the quality of inclusion studies. Statistical analysis was performed with heterogeneity test with *I*^2^ and chi-square tests. We summarized the mortality, prognosis of neurological function, and deep vein thrombosis (DVT) outcomes and presented as risk ratio (RR) or risk difference (RD) with a 95% CI.

**Results:**

Seven RCTs accounting for 1180 patients were included after meeting the inclusion criteria. Compared with placebo, the overall mortality of EPO-treated patients was significantly reduced (RR 0.68 [95% CI 0.50-0.93]; *p* = 0.02). EPO therapy did not improve neurological prognosis (RR 1.21 [95% CI 0.93-1.15]; *p* = 0.16) or increase the occurrence of DVT (RR 0.83 [95% CI 0.61–1.13]; *p* = 0.242), which showed no significant difference.

**Conclusions:**

The results showed that the administration of EPO may reduce the risk of mortality without enhancing the occurrence of DVT in TBI patients. However, the effect of EPO on neurological outcome remains indistinct. Through subgroup analysis, we demonstrated that the dose of EPO may be a potential factor affecting the heterogeneity in neurological function and that the follow-up duration may influence the stability of the result.

## 1. Introduction

Traumatic brain injury (TBI) is a leading cause of mortality and long-term disability, particularly affecting young people [1]. Expect for primary brain injury that occurs immediately following injury, secondary brain injury is highly heterogeneous in manifestation and multifactorial in etiology [2]. Posttraumatic brain injuries are mainly the result of compression, laceration, edema, hemorrhage, and ischemia [3]. Since it is almost impossible to reverse primary brain damage, many neuroprotective drugs have been widely studied for their protective effects by alleviating or limiting secondary brain injuries.

Anemia is frequent among in patients with TBI, which is considered to aggravate secondary brain damage and is related to an increased risk of poor prognosis [4–7]. The causes of anemia include hemorrhage due to bone tissue and soft tissue damage, gastrointestinal stress ulceration, and repetitive excessive blood sampling; it also includes traumatic changes in the internal environment resulting in red blood cell damage leading to intravascular hemolysis and dyserythropoiesis [8]. There is clinical consensus that allogenic red blood cell transfusion (RBCT) is needed for Hb less than 7  g/dL in critically ill patients with TBI [9, 10]. However, RBCT has corresponding risks and complications, the effects of which in moderately anemic patients remain controversial [7, 11]. Some studies have showed that transfusion was associated with worse outcomes while some studies have found no relationship between transfusion and outcome [6, 12, 13]. Moreover, some patients refuse to receive blood transfusions because of their religion or beliefs [14, 15]. Recombinant human erythropoietin and iron therapy were used for alternative effective therapy in anemia.

Erythropoietin (EPO) belongs to the type I cytokine superfamily as a glycoprotein of 165 amino acids, and it has been initially deemed as the hematopoiesis-regulating hormone [16]. In addition to treating anemia after TBI, EPO has a potential neuroprotective effect acting as biological antioxidant [17, 18]. A large number of preclinical studies have confirmed that exogenous EPO also has neuroprotective effects after traumatic brain injury through antiedematous, antioxidant, antiexcitotoxic, and anti-inflammatory mechanisms [3, 19]. In order to further researching the actual efficacy of EPO, some clinical trials assessed the effect and safety of EPO in patients with TBI. Among these trials, some studies have suggested that EPO plays an effective role in improving neurological outcome or/and decreasing the risk of mortality [20–23]. Nevertheless, other studies have reported that the use of EPO did not improve the prognosis of neurological function [24, 25] and may increase the risk of thrombosis [26]. To analyze these different findings, a meta-analysis study was performed and found that the treatment of EPO prevents death following TBI without causing adverse events, such as DVT, but its role in ameliorating neurological prognosis remains unclear. Consequently, this meta-analysis is aimed at collecting relevant RCTs to assess the effectiveness of EPO on mortality and neurological prognosis in the treatment of TBI to further explore the potential therapeutic effects of EPO.

## 2. Methods

### 2.1. Search Strategy and Selection Criteria

Included studies had to be all language publications of RCTs treated with any ESA in healthy patients with critically traumatic brain injury in spite of dose, frequency, and duration of treatment. The patient should be older than or equal to 15 years of age and should be treated in the hospital or the prehospital clinical setting. The following exclusion criteria retains: (1) non-RCTs; (2) pediatric patients (<15 y); (3) nonhuman models; (4) case studies, case series, letters, abstracts, and reviews; (5) non-English-language publications.

The searched databases include PubMed, OVID, and the Cochrane Library from inception until October 18, 2019. The term was researched erythropoietin, EPO, ESA, traumatic brain injury, and brain injury.

### 2.2. Data Extraction

Two investigators screened, assessed, and excluded articles separately according to the titles and abstracts by the inclusion and exclusion criteria. Full text downloaded for possible relevant titles and abstracts is assessed on the basis of inclusion and exclusion. Any divergence was solved with discussion and consensus. Some key data collected from eligible studies included the year of publication, first author, diagnosis, number of participants in each group, age and sex, therapeutic method, mortality, prognosis evaluation, and potential side effects. Functional outcomes were evaluated with Glasgow Outcome Scale (GOS) scores and the extended GOS (GOS-E) score.

### 2.3. Quality Assessment

We applied the Cochrane Collaboration's tool to independently evaluate the risk of bias on the basis of these content below: random sequence generation and allocation concealment (selection bias), blinding of participants and personnel (performance bias), blinding of outcome assessment (detection bias), incomplete outcome data (attrition bias), selective reporting (reporting bias), and other bias. According to the above evaluation items and types of bias for RCT, corresponding evaluation results are given, including low risk of bias, high risk of bias, and unclear.

### 2.4. Statistical Analysis

The primary outcome in this systematic review was investigated with mortality, functional neurological outcome, and deep vein thrombosis (DVT). Dichotomous variables are expressed as risk ratios (RRs) with 95% confidence intervals (CIs) while the mean difference (MD) and 95% CI were deemed as continuous variables. Heterogeneity was considered as having no (0% to 24.9%), mild (25% to 49.9%), moderate (50% to 74.9%), and significant (75% to 100%), respectively. Moreover, a chi-squared test was used, and *p* < 0.10 was considered statistically significant. Random effects model was applied when *I*^2^ value was larger than or equal to 50%, and the fixed effects model was used when *I*^2^ value was less than 50%. We performed sensitivity analyses in which single study was removed sequentially each time to confirm an overall approximate value of the remaining studies. Subgroup analysis was used for considering heterogeneous sources and influencing factors of result. Publication bias was assessed visually using Egger's or Begg's regression model in an analysis. Collected data was analyzed with Review Manager 5.3 and STATA software 15.1. All tests were 2-tailed, and *p* < 0.05 was regarded as statistically significant.

## 3. Results

**Figure 1:**
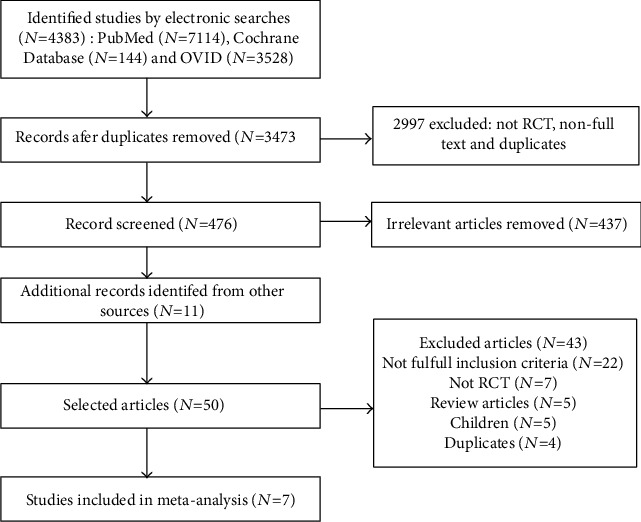
Flow diagram of literature retrieval for selecting included studies.

The result of identified articles and our search strategy is shown in the flow diagram (Figure 1). The electronic database searches found 4383 record in total, and a total of 3473 articles remained after excluding 910 duplicative records. By excluding non-RCT, non-full text, and duplicates, 476 articles were chosen. Then, two researchers independently screened according to the title and abstract of article and added 11 articles from other sources, cutting down to 50 studies. Forty-three studies were eventually excluded for incomplete inclusion criteria (*N* = 22), non-RCT (*N* = 7), review articles (*N* = 5), children (*N* = 5), and duplicates (*N* = 4). In conclusion, 7 RCTs were included in this meta-analysis.

The characteristics of the seven studies were summarized in Table 1. The sample size in each study ranged from 16 to 602, and the sum of patients was 1180. In these studies, the sum of participants in the EPO-treated group and placebo group was 604 and 576, respectively. The age range was greater than or equal to 15 years old, and the male accounted for the vast majority of the sex. The dose of EPO was between 2000 and 40,000 IU, and the first administration time ranged from 2 to 24 hours after admission.

### 3.1. Risk of Bias among Included Studies

**Figure 2:**
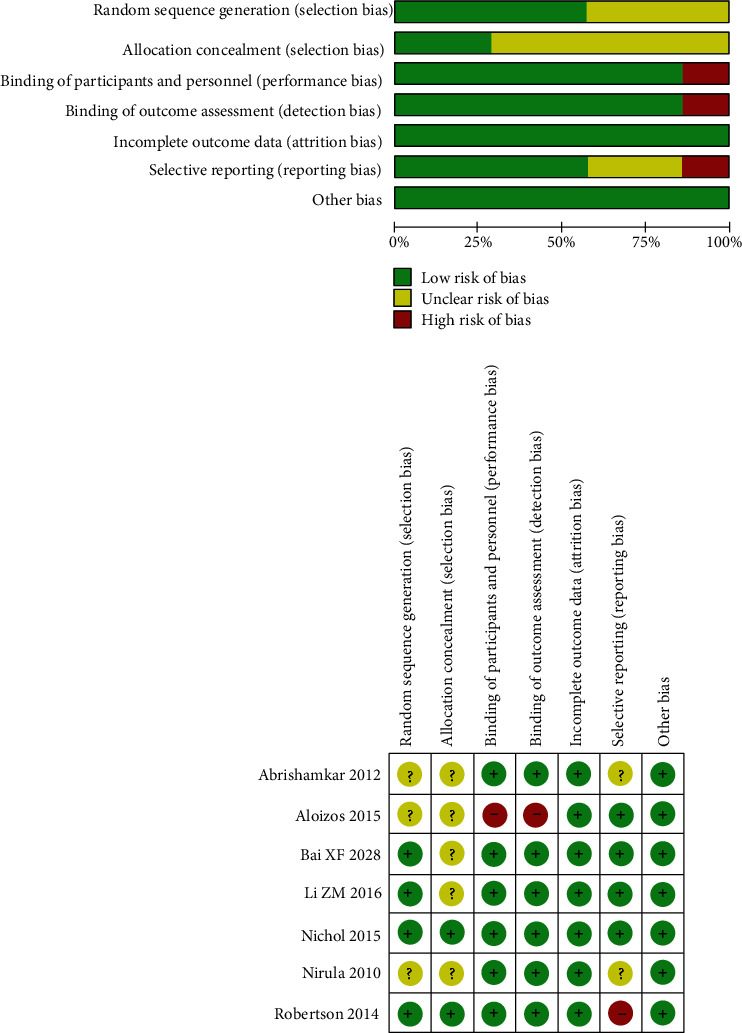
Risk of bias assessment for the include RCTs.

The risk of bias for the inclusion RCTs is summarized up in Figure 2. The random fashion was depicted in four studies, and allocation concealments were evaluated as uncertain risk in five studies. Except one study, six studies were considered as low risk in blinding of outcome assessment. All studies were deemed as low risk in incomplete outcome data and other bias. Selective reporting was assessed as unclear risk in two studies and high risk in one study.

### 3.2. Mortality

**Figure 3:**
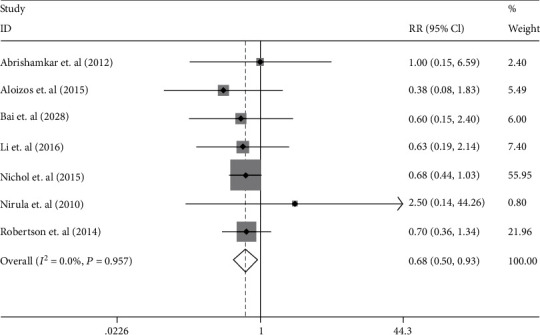
The forest plot of mortality in the patients with TBI treated with EPO in the fixed effects model.

Seven studies (involving 1161 patients) were analyzed with regard to the effect of EPO in mortality. We did not find any significant statistical evidence of heterogeneity between trials (*I*^2^ = 0%; *p* = 0.957). Therefore, the fixed model was adopted for analysis of Meta. The mortality rate in the EPO-treated groups and in the placebo groups was 10.8% (58 of 536) and 16.7% (81 of 486), respectively. The EPO treatment strategy was related to a significant reduction in mortality compared to the placebo group (RR 0.68 [95% CI 0.50-0.93]; *p* = 0.02) (see Figure 3).

### 3.3. Prognosis of Neurological Function

**Figure 4:**
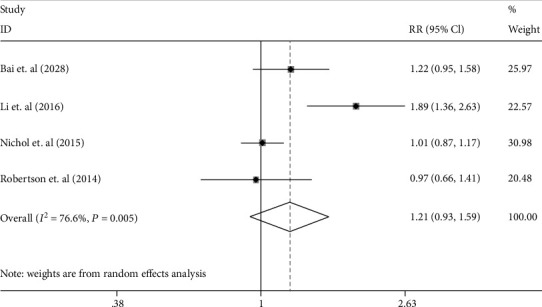
The forest plot of the improvement of the neurological function in the patients with TBI treated with EPO in random effects model.

The GOS score or the extended GOS (GOS-E) score was used for assessing functional neurological outcomes. Favorable neurological prognosis was deemed as a score of 4-5 in GOS and 5-8 in GOS-E. Four studies were included in this meta-analysis with 1043 patients. Favorable outcome occurred in 300 of 529 (56.7%) EPO-treated patients compared to 259 of 514 (50.3%) of untreated individuals. There was significantly high heterogeneity between trials (*I*^2^ = 76.6%; *p* = 0.005), and the random effects model was adopted. The results of the pooled analysis suggested that EPO had no statistical effect for improving neurological prognosis (RR 1.21 [95% CI 0.93–1.59], *p* = 0.16; see Figure 4).

### 3.4. DVT

**Figure 5:**
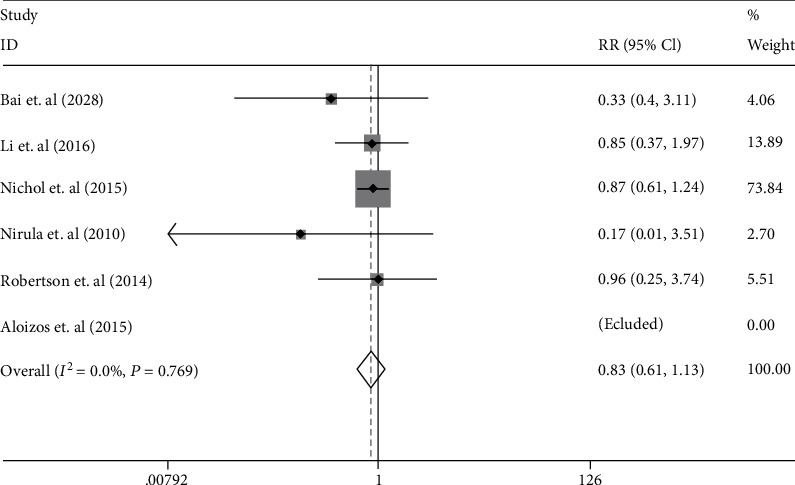
The forest plot of DVT incidence in the patients with TBI treated with EPO in fixed effects model.

Six studies were assessed in this analysis which included 62 of 577 (10.7%) patients in the EPO-treated group and 72 of 556 (12.9%) in the placebo group. There was no significant statistical heterogeneity between studies (*I*^2^ = 0%; *p* = 0.769), and the fixed effects model was applied. The results showed that EPO treatment in pooled analysis had no statistical effect for the incidence of DVT (RR 0.83 [95% CI 0.61–1.13], *p* = 0.242; Figure 5).

### 3.5. Sensitivity Analysis and Publication Bias

**Figure 6:**
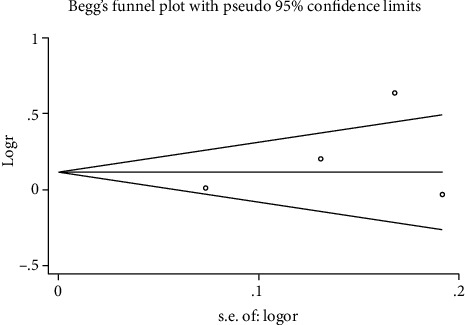
The funnel plot of Egger's test for the detection of publication bias on neurological prognosis.

The results of the heterogeneity of mortality and DVT were 0%, which suggested that all included studies were completely homogeneous. But heterogeneity of neurological outcomes was 76.6% and *p* < 0.05. The results of sensitivity analysis showed that the removal of any of the included studies had no statistical effect on neurological outcomes. Publication bias was assessed by Begg's test (pr > ∣*z* | = 0.734) and Egger's test (*p* > ∣*t* | = 0.630) which implied no visible asymmetry (see Figure 6). To analyze the sources of heterogeneity, we divided EPO dose into two groups for subgroup analysis, one group less than 10000 IU/dose and the other group more than or equal to 10000 IU/dose. We found that both subgroups were insignificant (RR 1.50 [95% CI 0.97–2.33], *p* = 0.069; RR 1.00 [95% CI 0.88–1.15], *p* = 0.953), but the heterogeneity of group of less than 10000 IU/dose was significant (*I*^2^ = 77.5%; *p* = 0.035), and there was no heterogeneity in group of more than or equal to 10000 IU/dose (*I*^2^ = 0%; *p* = 0.005).

## 4. Discussion

In this systematic review and meta-analysis of treatment of moderate to severe TBI with EPO, we found that EPO treatment was related to a reduced risk of mortality. Nevertheless, there was no significant statistical difference for improved neurological outcome and increased the incidence of DVT. With the above-mentioned search, we have newly included Bai and Gao 2018 as a new high-quality RCT, which can improve our analytical evidence of treatment outcomes. The results of our meta-analysis are compliance with the previous meta-analysis. But this result may not be fully consistent with the results of other clinical trials, such as Aloizos 2015. More high-quality, large-sample clinical trials are needed to further elucidate these conclusions.

In order to improve neurological prognosis and reduce mortality after TBI, many neuroprotective drugs were used to study the therapeutic effect on TBI patients in clinical study. Erythropoietin, as a pleiotropic cytokine produced in the kidney and central nervous system, has been regarded as potential neuroprotection [27]. With the further development of research, EPO therapy is of great significance for the treatment of TBI, mechanism of which is not entirely clear. Some studies suggest that the use of recombinant human erythropoietin may elevate Hb concentrations in critically ill patients, thus avoiding allogeneic blood transfusions [28]. Increased concentration of Hb after EPO treatment can restore hematocrit and blood oxygen-carrying capacity [29]. Except for treating anemia after TBI, EPO has a potential neuroprotective effect acting as biological antioxidant [17, 18]. A large number of preclinical studies have confirmed that exogenous EPO also has neuroprotective effects after traumatic brain injury through antiedematous, antioxidant, antiexcitotoxic, and anti-inflammatory mechanisms [3, 19]. Some studies have reported abnormally elevated iron and ferritin levels at the site of brain injury sites, which is independent of hemoglobin binding iron associated with blood leakage in the injury sites [30–32]. Excess iron causes neurotoxicity by promoting the formation of reactive oxygen species resulting in oxidative stress, lipid peroxidation, and ferroptosis. Iron chelators and antioxidants may be beneficial for brain injuries by decreasing iron content [33, 34]. EPO can decrease production of harmful free radicals by increasing erythropoiesis and iron utilization to play a neuroprotective role in TBIs [35].

However, when mortality improved after EPO treatment, the improvement in patients' poor prognosis has not been determined in this study, although death was one of the manifestations of poor prognosis. This question is the focus of many studies. Some animal experiments and clinical trials with neonates for EPO administration have also provided important information about the effect of EPO. Peng et al. [36] have found that EPO might be beneficial in decreasing lesion volume and improving neurobehavioral outcome in experimental animal models of TBI in meta-analysis. Razak and Hussain [29] have showed that the use of EPO in neonates with perinatal hypoxic-ischemic encephalopathy may reduce the risk of mortality, cerebral palsy, cognitive impairment, and brain injury in meta-analysis. Corwin et al. [28] have reported that the treatment of EPO may reduce 29-day mortality in critically ill patients with trauma and may be related with a significant increase in the occurrence of thrombotic events. Luchette et al. [37] have observed that there was no difference in function outcomes or safety in anemic, critically ill, trauma patients treated with epoetin alfa (1.2 g/dL) compared to placebo (0.9 g/dL). Talving et al. [38] have showed that erythropoietin administration had a significant survival advantage and did not increase morbidity in TBI patients. There was no statistical difference in the occurrence of major in-hospital complications including DVT embolism comparing with 2 study cohorts. Some RCTs also suggested that EPO therapy significantly improved long-term neurological prognosis and reduce some adverse events in patients after stroke [39, 40]. Due to strict inclusion requirements, some clinical studies were not included in this review, but they still have guiding significance for the study of EPO therapy. So, combined with previous study, these results indicated that EPO may be potentially effective treatment for improving neurological outcome. However, in this review, four RCTs were involved in the study of neurological outcome, and only one RCT suggested that EPO could significantly improve neurological function. These reasons may include the time of first administration, the differences in EPO administration dosages, and the complexity of the condition in patient with TBI. Therefore, RCTs requiring more large sample and multicenter are needed to confirm the effects of drug dosage, duration of action, and long-term prognosis of neurological function.

In the selected studies, we found that the follow-up duration exists significant differences which range from 2 weeks to 6 months. In Australian EPO-TBI clinical trial, Skrifvars et al. [41] have found that the mortality of EPO group was not significantly different from that of the control group at different follow-up duration. To evaluate the effect of follow-up duration on result, we can divide it into less than 3 months and more than or equal to 3 months to perform subgroup analysis according to the observation time. We thought that there may be more reliable conclusions in subgroup of more than or equal to 3 months compared with subgroup of less than 3 months on mortality (RR 0.66 [95% CI 0.47–0.92]; *p* = 0.014 vs. RR 0.87 [95% CI 0.32–2.39]; *p* = 0.786) and DVT (RR 0.87 [95% CI 0.63–1.21]; *p* = 0.396 vs. RR 0.27 [95% CI 0.04–1.58]; *p* = 0.146).

Moreover, we proposed a different point of view. Li et al. asserted that both random sequence generation and allocation concealment are low risk in Nirula et al. 2010, and attrition bias is unclear as well as other bias is high risk in Abrishamkar et al. 2012. Considering that they only performed a random assignment without specific random scheme, we thought that selection bias in Nirula et al. 2010 is not unclear. Moreover, we did not find any missing description or other bias in Abrishamkar et al. 2012.

There are several limitations in this study. First, we did not investigate the effect of dose and frequency of EPO administration in TBI patients, which might influence the effect of EPO treatment. Therefore, more subgroup analysis is needed to assess its impact. Second, our meta-analysis failed to obtain enough personal data for patients with TBIs to evaluate the impact of EPO and may neglect the potential factors for assessing the effects of mortality, neurological outcomes, and adverse reactions. The sex ratio of each study was different, and sex hormone differences may affect the results. The impact of TBI severity on outcomes was not assessed. The time difference of judging death or poor prognosis of patients is large, which may affect the judgment of results due to the early termination of observation. Finally, our findings are limited to the inclusion of published data as the study of negative results is unlikely to be published.

## 5. Conclusions

Our studies showed that the administration of EPO may reduce the risk of mortality without increasing the incidence of DVT in TBIs patients, although the effect of EPO on neurological outcome remains indistinct. Through subgroup analysis, we demonstrated that the follow-up duration may be a potential factor influence the stability of the result.

## Figures and Tables

**Table 1:** Characteristics of included studies.

Author & year	Diagnosis	Intervention	Number of participants (EPO/placebo)	Age (yrs, EPO/placebo)	Sex (males (females) in EPO/placebo)	Outcome measure	Outcomes measure time
Bai and Gao 2018	Severe TBI	6000 IU, within 2 h, on days 3, 5, 10, and 15	120 (60/60)	44.5 ± 11.4/43.1 ± 10.9	41 (19)/44 (16)	GOS scores & mortality & adverse events	10 wks
Li et al. 2016	Severe TBI	100 units/kg, within 6 h, on days 3, 6, 9, and 12	146 (75/71)	43.4 ± 10.1/41.1 ± 9.6	49 (26)/41 (30)	GOS scores & mortality & adverse events	3 mos
Nichol et al. 2015	Moderate or severe TBI	40,000 IU, within 24 h, weekly for a max of 3 doses	602 (305/297)	30.5 (22.4-47.5)/30.5 (22.9-48.3)	256 (49)/246 (52)	GOS-E score & mortality & adverse events	6 mos
Aloizos et al. 2015	Severe TBI	10000 IU, within 6 h, 7 consecutive days	42 (24/18)	29.4 ± 1.3/46.5 ± 4.5	23 (19)/16 (2)	GOS-E score & adverse events	6 mos
Robertson et al. 2014	Severe TBI	500 IU/kg, 1st dose within 6 h, weekly for 2 more weeks	200 (102/98)	31.5 (23-48)/30.0 (22-44)	89 (12)/84 (14)	GOS, DRS score & adverse events	6 mos
Abrishamkar et al. 2012	Severe TBI with DAI	2000 U, on days 2, 4, 6, 8, and 10	54 (27/27)	25.2 ± 5.4/27.3 ± 4.8	27 (0)/27 (0)	GOS & mortality	2 wks
Nirula et al. 2010	Moderate or severe TBI	40,000 units within 6 h	16 (11/5)	35 ± 19/40 ± 26	8 (3)/3 (2)	Serum NSE, S-100B, ICP values, mortality & adverse events	5 d

DRS: Disability Rating Scale; NSE: neuron-specific enolase; DAI: diffuse axonal injury; ICP: intracranial pressure. Age was presented as median (IQR) or mean.

## Data Availability

The data supporting this meta-analysis are from previously reported studies and datasets, which have been cited.
